# Supplementary material for: Exploring the Nexus of Climate Change and Substance Abuse: A Scoping Review
Source: Int J Environ Res Public Health. 2024 Jul 9;21(7):896. doi: 10.3390/ijerph21070896 (PMC11277026; doi:10.3390/ijerph21070896)
Supplement: Supplementary file 1 [file ijerph-21-00896-s001.zip › ijerph-3025731-supplementary.pdf]

Table S1. Summary table regarding the selected works within the scope of the scoping review.

Reference	Year	Type of Study	Study Aim	Drugs Cited	Sample Size	Results	Conclusion
Chang, H. H., et al [23]	2023	observational study	The study aims to investigate the connections between daily ambient temperature and emergency department visits related to specific drug use and overdoses in California across a 15-year focusing on amphetamine, cocaine, and opioids. It seeks to understand the connection between the stimulants and opioid to short-term temperature increments and to consider alternative measures to reduce heat exposure.	Amphetamine, cocaine, opioids.	3.4 million drug-related emergency department (ED) visits.	The study shows that Higher temperatures significantly increase emergency department visits for amphetamine, cocaine, and opioid use and overdoses, as revealed by over 3.4 million cases in California from 2005 to 2019.	The study reveals that individuals using stimulants and opioids are more likely to reach emergency care during hotter temperatures, especially with amphetamines, cocaine, and opioids. It highlights how heat influences drug use patterns and pointed out the urge for more research on healthcare responses to heat-related emergencies, and fair climate health policies.
Kilbourne EM [31]	1998	ecological study	The aim of the study is to explore the correlation between heat waves and fatalities resulting from cocaine overdose. By analysing the data from the New York City Medical Examiner's Office, Marzuk and colleagues aimed to present evidence indicating a notable rise in the risk of death due to cocaine overdose during periods of high temperatures. The researchers sought to identify instances of deaths attributed to cocaine overdose and to illustrate that such occurrences are more prevalent during hotter weather in New York City.	Cocaine<br>Opioids	N/A	The study finds a strong correlation between heat waves and increased cocaine overdose deaths in New York, with a specific heat-related effect on cocaine overdoses.	The study emphasizes a distinct association between heatwaves and elevated cocaine overdose fatalities, separate from other drug-related deaths. The results indicate that heat exacerbates the risk of cocaine overdose specifically, unlike opioids. It suggests a potential dose-response correlation between heat and these fatalities, underscoring the necessity for additional research to comprehensively grasp this connection.
Cusack L.et.al.[14]	2011	qualitative discussion paper	The study aims to examine the clinical implications of negative health outcomes during heatwaves for individuals with mental health disorders, substance abuse, and prescribed medications such as lithium, various neuroleptic, and anticholinergic drugs.	Opioids<br>Alcohol<br>Diazepam<br>Amphetamines<br>others	N/A<br>(it is a qualitative study)	The document explores how heatwaves exacerbate health risks for people with mental health issues, substance abuse, and medications. It highlights the dangers of heat-related illnesses such as heat	The research concludes that heatwaves present an elevated risk for individuals with mental health disorders, substance abuse, and those taking certain medications. It advocates for heightened health surveillance during heatwaves

						stroke and exhaustion, and how to prevent health complications.	and the implementation of strategies to prevent health complications.
Marzuk PM et. Al.[25]	1998	retrospective review	The aim of the study was to investigate the correlation between mortality due to unintentional cocaine overdose and hot weather conditions in New York City.	Antipsychotic medications<br>Antidepressant medications<br>Antiparkinsonian and antihistaminic medications<br>Psychostimulants & sympathomimetic drugs<br>Phencyclidine	The study's sample size included all fatal unintentional cocaine overdoses from 1993 through 1995 (n = 2008), in addition to other comparison groups.	The study found that when temperatures reached 31.1°C or higher, the daily number of cocaine overdose deaths increased by 33%. However, opiate overdose deaths did not show a significant increase on hot days. Furthermore, the proportions of cocaine-positive toxicology results in other overdose deaths, homicides, and traffic fatalities remained consistent regardless of temperature. This suggests that high temperatures are associated with a significant increase in cocaine overdose deaths, rather than simply increased cocaine use among the population.	The study's conclusion highlights that higher ambient temperatures significantly raise mortality rates from cocaine overdose. The 33% increase in daily cocaine overdose deaths on hot days suggests heightened risks associated with cocaine during warmer weather, regardless of changes in its use among the population. This underscores the increased danger of cocaine during elevated temperatures and emphasizes the importance of awareness and preventive measures.
Reser, J.P et. Al.[30]	2014-2019	quantitative study	The study aimed to review articles published from 2014 to 2019, exploring how researchers investigated the importance of personal experiences with environmental changes associated with climate change. Additionally, it sought to understand how individuals' own experiences with climate-related changes influence their level of concern about climate issues.	N/A	15	From 2014 to 2019, most studies focused on assessing individuals' exposure to extreme weather events and their perceptions of climate change effects. These studies revealed that various weather events influenced people's thoughts, emotions, and actions regard-	Researchers advocate for long-term studies to comprehend how these experiences evolve over time. They propose that all scientists adopt uniform methodologies while maintaining their unique perspectives. Furthermore, they suggest that research should be more transparent to facilitate the integration of social sciences with climate change science, aiding in a

						ing climate change. Factors such as political, environmental, and personal beliefs significantly influenced these perceptions.	better understanding among researchers from different fields.
Parks RM et al.[26]	1995-2014	observational study	The study aimed to investigate the relationship between daily temperatures and hospital admissions for issues related to alcohol or various drugs, including cannabis, cocaine, opioids, and sedatives. Additionally, it examined how this correlation varied depending on individuals' geographical location, age, gender, and social vulnerability.	cannabiscocaine, opioids, sedatives	671,625 complete hospital visit records for alcohol-related disorders and 721,469 complete hospital visit records for substance-related disorders. All data collected are refereed to New York State	The study found that as temperatures rise, there is a corresponding increase in hospital admissions for alcohol and drug-related issues. Specifically, there is a 24.6% rise in hospital visits for alcohol-related problems and a 37.7% increase for drug-related problems during hotter periods. This trend applies to individuals of all ages, genders, and social backgrounds. Interestingly, outside of New York City, the correlation between hot weather and drug problems is even more pronounced.	The study indicates that during hot weather, there is an increase in hospital admissions due to alcohol and drug-related issues, and this trend may worsen with climate change. It suggests the necessity of enhancing support and healthcare services to mitigate the rise in hospital visits attributed to heat-related factors. This underscores the severity of climate change on public health, emphasizing the urgency for action.
Hensel M et.al.[22]	2021	prospective observational study	The aim of the study was to test the hypothesis that severe acute poisoning by alcohol and drugs is more frequent at higher rather than at lower ambient temperatures.	alcohol, opioids, sedatives, hypnotics, multiple drugs, volatile solvents, psychoactive substances.	1,535 patients	The study's findings revealed that among 1,535 patients, cases of severe acute alcohol or drug poisoning, characterized by symptoms such as loss of consciousness, hypotension, and impaired respiratory function, were treated. It was observed that compared to mild temperatures (10-20°C), the incidence of poisoning	The study discovered that during hotter weather, there is an increase in emergencies related to severe alcohol and drug poisoning. However, the severity of these emergencies does not necessarily escalate during hotter temperatures. These findings may vary in other regions due to differences in how various areas manage hot weather conditions. Nonetheless, it is crucial to comprehend the local impact of temperature on health and

						increased during higher temperatures and decreased during lower temperatures. However, no significant correlation was detected between the severity of emergencies and temperature.	implement systems to alert individuals about health risks during hot weather.
Aarde SM et.al.[24]	2017	Experimental study	The aim of the study is to investigate the influence of elevated ambient temperatures on the acquisition of 3,4-methylenedioxymethamphetamine (MDMA) self-administration among the test subjects. This entails examining differences in MDMA intake under varied ambient temperature conditions and assessing the effects of temperature on body temperature and activity levels during both the acquisition and maintenance phases of the drug self-administration study.	MDMA cocaine, MDPV entactogen-class stimulants.	39	The study revealed variations in MDMA intake, body temperature, and activity levels between the Hot-trained and Cold-trained groups across different phases of the drug self-administration study conducted under varying temperature conditions. During the initial phase, intake levels increased in the Hot group, while body temperature decreased across sessions for all groups. Activity levels exhibited differences between groups across various study phases. Additionally, body temperature consistently remained lower in the Hot-trained group compared to the Cold-trained group throughout the study.	The study suggests that subjects tend to consume more MDMA in warmer weather, which could potentially influence their future intake based on past experiences with the drug at various temperatures. Test subjects may increase MDMA consumption during warmer temperatures because their brains perceive greater reward sensations in such conditions. Additionally, the study indicates that subjects develop a tolerance to MDMA's effects on their body temperature over time.
Hudson K et.al.[27]	2021	Review	The aim of the study is to review recent literature on the effects of climate change on child and adolescent mental health and to discuss treatment and engagement by clinicians	antipsychotics	2000	The findings of the article suggest that climate change has various impacts on the mental health of children and teenagers.	The conclusion of the article emphasizes the significant impact of climate change on children's mental health. It underscores the im-

						It serves as both a social and ecological factor influencing health, exacerbates existing threats, and induces trauma and distress. Even individual extreme weather events can significantly negatively impact mental health, while ongoing climate changes also affect mental well-being. Awareness of the climate crisis can lead to emotional distress. Moreover, climate change exacerbates pre-existing health disparities and affects communities disparately based on factors such as race, class, and geographical location.	portance for mental health professionals to address this issue seriously. The conclusion offers suggestions measurements that can be taken by doctors, such as incorporating climate change into training, addressing mental health issues related to climate change, assisting children in coping with climate-related stressors, and supporting efforts to address climate issues. Additionally, it discusses the crucial role that doctors play in advocating for long-term solutions and collaborating with young activists who are passionate about addressing climate change.
Hrabok M et.al.[15]	2020	Qualitative study	The study wants to talk about how climate change affects mental health, especially after natural disasters. It also wants to look at what makes mental health problems worse or better after disasters.	Alcohol	N/A	The study's results are as follows:<br>1. Post-Traumatic Stress Disorder (PTSD): PTSD is frequently observed after disasters, with symptoms appearing following events such as wildfires, earthquakes, and hurricanes. Its prevalence is influenced by factors such as gender, age, socioeconomic status, severity of exposure, and post-disaster effects.<br>2. Depression: Depression	The conclusion of the study discusses several important points:<br>1. Mental Health Effects: Climate change can impact mental health, leading to anxiety and depression, especially following traumatic events such as disasters.<br>2. Vulnerability Factors: Certain groups, including women, individuals with low income, and those with pre-existing mental health conditions, are more susceptible to experiencing mental health issues after disasters.<br>3. Children and Youth: Young people are particularly vulnerable due

						may persist following extreme weather events, associated with phenomena related to climate change. Suicidal tendencies may be affected by climate-related changes such as air pollution and rising temperatures.<br>3. Anxiety: Rates of anxiety increase following acute weather events, particularly associated with extreme occurrences like forest fires and heatwaves.<br>4. Alcohol Consumption: Alcohol consumption often increases post-disaster due to factors such as exposure or PTSD.	to their social and familiar circumstances, as well as concerns regarding the long-term consequences of climate change.<br>4. Community Strategies: Communities should implement strategies such as enhancing access to mental health care.<br>5. Future Research Needs: Further research should concentrate on understanding how climate change influences mental health.
Ezell JM et.al.[16]	2023	Qualitative study	The study aims to investigate the potential impacts of climate change on individuals with opioid use disorder. It seeks to understand why certain regions are more affected by climate change than others and to devise strategies to mitigate these effects.	Opioids<br>Methamphetamine<br>Crack<br>Cocaine<br>GHB/GBL<br>Ketamine<br>PCP<br>Heroin<br>ecstasy	N/A	The document examines the potential impacts of climate change on individuals coping with opioid addiction and provides strategies to support them. It discusses how factors such as social issues and drug contamination can increase the vulnerability of these individuals to the effects of climate change. It underscores the necessity of adopting a comprehensive approach to address both	The study emphasizes the importance of addressing the opioid crisis and climate change concurrently, especially for individuals struggling with opioid addiction. It highlights how climate change can exacerbate drug-related issues and contribute to widening health disparities. The study recommends various actions including enhancing drug safety measures, expanding addiction treatment and support services, providing housing assistance, and educating individuals with addiction about climate change

						the opioid crisis and climate change.	
Ryus Cet.al.[28]	2021	Qualitative study	The aim of the study presented in the article is to underscore the particular susceptibility of individuals with opioid use disorder to heat-related illnesses. This objective is designed to prompt additional research, provide insights to scientists and healthcare providers, and illuminate the potential connections between the opioid crisis and climate change as factors contributing to heightened morbidity and mortality.	Fentanyl<br>Heroin<br>Benzodiazepines	N/A	The study's findings reveal a developing correlation between the opioid crisis and climate change. It underscores the heightened risk of complications and mortality among patients with opioid use disorder amid rising temperatures and heatwaves. The cases examined in the study underscore the susceptibility of individuals with opioid use disorders to heat-related illnesses, underscoring the synergistic interplay between opioid use and extreme heat conditions, which exacerbates medical complications, morbidity, and mortality.	The study concludes that individuals with opioid addiction are at increased risk of severe health issues or death due to extreme heat. It suggests collaborative efforts among communities, healthcare professionals, and researchers to address both opioid addiction and heat-related illnesses. Emphasizing the importance of preparedness during hot weather, the study recommends that emergency care providers be equipped to address both heat-related issues and opioid overdoses. Lastly, it underscores the necessity for further research to enhance understanding and treatment of the relationship between opioid addiction and heat exposure.
Cusack L et.al.[14]	2011	Discussion paper<br>Qualitative study	The aim of the study is to examine the clinical implications of adverse health outcomes resulting from heatwaves for individuals with mental health disorders, substance abuse is-sues, and those taking specific prescribed medications. This involves investigating the impact of heat-waves on individuals with comorbidities, including the physiological aspects of heat stress, heat regulation, and the effects of alcohol and other drugs.	amphetamines, methamphetamine<br>Cocaine<br>MDMA<br>SSRIs<br>others	N/A	The study aimed to examine the clinical implications of adverse health outcomes during heatwaves for individuals with mental health disorders, substance misuse issues, and those taking prescribed medications. The document outlined data sources, background information, discussions on heat stress and regula-	The study found that heatwaves can exacerbate the health conditions of individuals with mental health issues or substance abuse problems, potentially leading to severe illness or increased risk of death. The study emphasizes the collective responsibility of mental health workers, nurses, and doctors to ensure the safety of these individuals during periods of hot weather. Recommendations include monitoring their health sta-

						tion, and practical strategies to mitigate the impact of heatwaves on vulnerable populations.	tus, educating them and their families about heat safety measures, providing access to cool environments if necessary, and implementing special measures to safeguard them during hot weather conditions. Practical strategies are essential to mitigate the impact on healthcare services during heatwaves
Page LA et.al.[29]	2012	observational study	The aim is to estimate the risk posed by high ambient temperatures on patients with psychosis, dementia, and substance misuse.	Antipsychotics Antidepressants Hypnotics Anxiolytics Alcohol	22.562	The study revealed that patients with mental illness exhibited an overall increase in the risk of death of 4.9% per 1.8°C rise in temperature. Among these patients, younger individuals and those primarily diagnosed with substance misuse displayed the highest mortality risk. Consequently, the study concluded that the heightened risk of death during hot weather among patients with psychosis, dementia, and substance misuse carries significant implications for public health strategies during heatwaves.	The conclusion of the study suggests that patients with psychosis, dementia, and substance misuse exhibit an elevated risk of mortality during hot weather. Additionally, the study identified that younger patients and those primarily grappling with substance misuse are at the greatest risk of death during heatwaves. Therefore, it is imperative for public health strategies to address the protection of individuals with mental health issues during periods of elevated temperatures.
